# A molecular switch at the yeast mitoribosomal tunnel exit controls cytochrome *b* synthesis

**DOI:** 10.1093/nar/gkaf634

**Published:** 2025-07-10

**Authors:** Andreas Carlström, Joseph B Bridgers, Mary Couvillion, Abeer Prakash Singh, Ignasi Forné, Axel Imhof, L Stirling Churchman, Martin Ott

**Affiliations:** Department of Biochemistry and Biophysics, Stockholm University, Stockholm 106 91, Sweden; Department of Medical Biochemistry and Cell Biology, Institute of Biomedicine, University of Gothenburg, Gothenburg 405 30, Sweden; Department of Genetics, Blavatnik Institute, Harvard Medical School, Boston, MA 02115, United States; Department of Genetics, Blavatnik Institute, Harvard Medical School, Boston, MA 02115, United States; Department of Medical Biochemistry and Cell Biology, Institute of Biomedicine, University of Gothenburg, Gothenburg 405 30, Sweden; Biomedical Center Munich, Faculty of Medicine, Ludwig Maximilian University of Munich, Planegg, Martinsried 82152, Germany; Biomedical Center Munich, Faculty of Medicine, Ludwig Maximilian University of Munich, Planegg, Martinsried 82152, Germany; Department of Genetics, Blavatnik Institute, Harvard Medical School, Boston, MA 02115, United States; Department of Biochemistry and Biophysics, Stockholm University, Stockholm 106 91, Sweden; Department of Medical Biochemistry and Cell Biology, Institute of Biomedicine, University of Gothenburg, Gothenburg 405 30, Sweden

## Abstract

Mitochondrial gene expression needs to be balanced with cytosolic translation to produce oxidative phosphorylation complexes. In yeast, translational feedback loops involving lowly expressed proteins called translational activators help to achieve this balance. Synthesis of cytochrome *b* (Cyt*b* or *COB*), a core subunit of complex III in the respiratory chain, is controlled by three translational activators and the assembly factor Cbp3–Cbp6. However, the molecular interface between the *COB* translational feedback loop and complex III assembly is yet unknown. Here, using protein-proximity mapping combined with selective mitoribosome profiling, we reveal the components and dynamics of the molecular switch controlling *COB* translation. Specifically, we demonstrate that Mrx4, a previously uncharacterized ligand of the mitoribosomal polypeptide tunnel exit, interacts with either the assembly factor Cbp3–Cbp6 or with the translational activator Cbs2. These reciprocal interactions determine whether the translational activator complex with bound *COB* messenger RNA (mRNA) can interact with the mRNA channel exit on the small ribosomal subunit for translation initiation. Organization of the feedback loop at the tunnel exit therefore orchestrates mitochondrial translation with respiratory chain biogenesis.

## Graphical Abstract

**Graphical Abstract:**
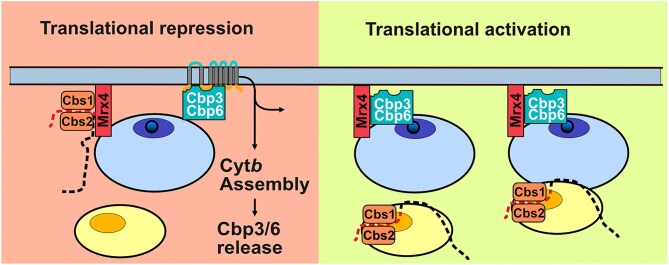

## Introduction

Organellar protein synthesis is a remnant of the endosymbiotic origin of mitochondria and chloroplasts. Proteins encoded in mitochondrial DNA (mtDNA) make up a small, but critical fraction of large multi-subunit assemblies, namely mitochondrial ribosomes (mitoribosomes) and the complexes driving oxidative phosphorylation (OXPHOS). Most subunits of the mitoribosome and OXPHOS are encoded in the nuclear genome, synthesized in the cytosol, and imported into mitochondria, where they are assembled with the mitochondrial translation products. The dual genetic origin of OXPHOS subunits and their assembly into complexes with defined stoichiometry necessitates the coordination of both genetic systems for a balanced translational output [1, 2]. Expression of nuclear-encoded genes is tightly regulated by transcription factors, but organellar gene expression can also be modulated [3]. While chloroplast gene expression employs a rather wide variety of options [4–6], accumulation of mitochondrial translation products is mainly adjusted through translational control [7–9] or through degradation of proteins produced in excess [10–12].

In the baker’s yeast, *Saccharomyces cerevisiae*, translational control of a subset of mitochondrial messenger RNAs (mRNAs) is achieved through feedback regulation by translational activators (TAs) [7, 9, 13]. By this, translational output is adjusted to levels that allow efficient assembly of the newly synthesized proteins. Here, the case of mitochondrial encoded cytochrome *b* (Cyt*b*) is best understood. Cyt*b* is the central subunit of the respiratory chain complex III and a highly hydrophobic membrane protein that participates in electron transport via its two heme *b* cofactors. Cyt*b* is assembled with nine nuclear-encoded proteins to form complex III [14], which in turn associates with complex IV to form respiratory supercomplexes [15, 16].

Synthesis of Cyt*b* by mitoribosomes depends on a set of TAs, proteins that bind to the Cyt*b*-encoding mRNA (*COB*) to support translation. How exactly the TAs of *COB* mRNA (Cbs1, Cbs2, and Cbp1) work to activate translation of their client mRNA is currently not well understood. Proximity mapping has shown that *COB* TAs are dual localized on the surface of the mitoribosome, binding close to the mRNA channel exit (MCE) on the small ribosomal subunit (SSU) to aid translation initiation of the mRNA, and relocating to the polypeptide tunnel exit (PTE) of the large ribosomal subunit (LSU) upon translational repression [17, 18]. The localization of *COB* TAs to alternative sub-ribosomal sites coincides with spatial dynamics of the assembly factor Cbp3–Cbp6. This heterodimer localizes to the mitoribosomal PTE [18, 19], where it is able to interact with the newly synthesized Cyt*b*. Binding to Cyt*b* releases Cbp3–Cbp6 from the mitoribosome [19], after which the Cyt*b*–Cbp3–Cbp6 complex is channeled into the assembly line of complex III [20, 21].

Localization of Cbp3–Cbp6 to the PTE is the key detection step of the *COB* mRNA feedback loop, which subsequently leads to translation activation. Its PTE association reports that the complex is available to chaperone and assemble newly synthesized Cyt*b*. In the event of stalled assembly, for example due to a deficiency in nuclear-encoded subunits, Cbp3–Cbp6 is sequestered in assembly intermediates and cannot activate translation. To this end, Cbp3–Cbp6 and the TA complex formed by Cbs1, Cbs2, and the *COB* mRNA localize to the PTE in a mutually exclusive manner [17], but how these reciprocal interactions are orchestrated and how these presumable dynamic interactions are conveyed to translational regulation remains unclear.

Here, we reveal the molecular mechanism underlying this translational feedback loop, which hinges on the dynamic, context-dependent interactions of TAs and Cbp3–Cbp6 at strategic sites on the mitoribosome. At the center of this regulatory circuit is Mrx4, a novel ligand of the mitoribosomal PTE that acts as a molecular switch. Mrx4 orchestrates the feedback loop by providing a mutually exclusive binding site for either the *COB* TA Cbs2 or the assembly factor Cbp3–Cbp6. This reciprocal binding to Mrx4 toggles between repression and activation of *COB* mRNA translation. Mrx4 represses translation of *COB* through sequestration of the TA complex at the PTE. Following the binding of Cbp3–Cbp6 to Mrx4, the TA complex is released from the PTE and relocates to the MCE for translation initiation. Thus, Mrx4 directly couples complex III assembly status to *COB* translation, ensuring precise coordination between mitochondrial and nuclear gene expression.

## Materials and methods

### Yeast strains used in the study

All strains in this study were isogenic to *S. cerevisiae* strains W303 *MAT*a {*leu2-3112 can1-100 trp1-1 ura3-1 ade2-1 his3-11*,*15*} or S288c [1] and are listed in Supplementary Table S1. Strains with modified mitochondrial genomes were created in previous studies [20, 22]. Chromosomal open reading frames (ORFs) were modified via homologous recombination according to [23]. ORFs were disrupted by insertion of *URA3*,*LEU2*,*KanMX4*, and *hphMX6* selection cassettes. FLAG, ALFA, and BirA* C-terminal epitope tags were added by replacing the stop codon of endogenous ORFs with the respective tag sequence followed by *HIS3* or *TRP1* selection cassettes. After transformation, genomic modifications were confirmed by growth on selection media and control polymerase chain reaction. For quantitative experimental approaches, three to five positively tested clones were applied to rule out congenic variations.

### Media and culturing conditions

Strains were grown at 30°C and 170 rpm in either full media containing 1% yeast extract, 2% peptone, and 2% glucose (YPD), galactose (YPGal) or glycerol (YPG) as indicated, or synthetic complete (SC) media, consisting of 0.17% yeast nitrogen base, 0.5% (NH_4_)_2_SO_4_, and 30 mg/l of all amino acids (except 80 mg/l histidine and 200 mg/l leucine) and 30 mg/l adenine and uracil with 2% galactose (SGal).

### Mitochondrial isolation

Yeast cells were grown overnight in YPG or YPGal to exponential phase (OD_600_ = 1–3) before harvesting through centrifugation at 3000 × *g* for 5 min. Cells were washed with distilled water, resuspended in MP1 buffer [2 ml/g wet weight of 0.1 M Tris-base, 10 mM dithiothreitol (DTT) according to 2 ml/g wet weight], and incubated for 10 min at 30°C before being washed with 1.2 M sorbitol, resuspended in MP2 buffer [6.7 ml/g wet weight of 20 mM KPi, pH 7.4, 0.6 M sorbitol, and 3 mg/g wet weight of zymolyase (Seikagaku Biobusiness, Tokyo, Japan)], and incubated at 30°C for 1 h to digest the yeast cell wall. Spheroplasts were harvested at 3000 × *g* for 5 min at 4°C, resuspended in MP3 buffer [13.4 ml/g wet weight of 0.6 M sorbitol, 10 mM Tris, pH 7.4, 1 mM ethylenediaminetetraacetic acid (EDTA), and 1 mM phenylmethylsulfonyl fluoride (PMSF)], and homogenized in 2 × 10 strokes using a tight-fitting homogenizer (Sartorius Stedim Biotech S.A., France). The homogenate was centrifuged two times at 3000 × *g* for 5 min at 4°C before mitochondria were harvested by centrifugation at 15 000 × *g* for 15 min at 4°C. The mitochondrial pellet was resuspended in SH buffer (0.6 M sorbitol and 20 mM HEPES, pH 7.4) to a final concentration of 10 mg/ml and frozen in liquid nitrogen before storage at −80°C.

### Western blotting

Proteins were separated on 16%/0.2% acrylamide/bis-acrylamide gels and transferred to nitrocellulose membranes. Transferred proteins were visualized by Ponceau staining (0.02% Ponceau red in 5% acetic acid), then blocked by incubation with 5% skim milk in TBS (Tris–HCl, pH 7.4, 150 mM NaCl) for 30 min, after which the proteins were detected by specific antibodies, namely Cbp3, Cbp6, Bca1, Tom70, Qcr7, Arg8 [19], bl31m [24], Aco1 [20], and uS5m [25]. FLAG antibody was purchased from Sigma–Aldrich (Merck). Antibodies against Mrx4 were obtained by immunizing rabbits with purified recombinant Mrx4 lacking the N-terminal mitochondrial targeting signal and the C-terminal transmembrane domain.

### Carbonate extraction and protease protection assay

For the carbonate extraction assay, 100 μg of isolated mitochondria was resuspended in 0.1 M Na_2_CO_3_ (treatment) or 0.1 M NaCl (control), incubated for 30 min on ice, and centrifuged at 100 000 x *g* for 30 min at 4°C. Membrane and soluble fractions were TCA precipitated and analyzed by sodium dodecyl sulfate–polyacrylamide gel electrophoresis (SDS–PAGE) and western blotting. For the protease protection assay, 100 μg of isolated mitochondria was incubated in SH buffer (whole mitochondria), 20 mM HEPES/KOH (pH 7.4) (mitoplasts), or lysis buffer (SH buffer with 0.2% Triton X-100) for 30 min on ice. Proteinase K was added to a final concentration of 10 μg/ml and incubated for 20 min on ice. PMSF was added to a final concentration of 1 mM before intact mitochondria and mitoplasts were centrifuged at 25 000 x *g* for 10 min at 4°C. Supernatants and pellets were TCA precipitated and analyzed by SDS–PAGE and western blotting.

### *In vivo* and in organello radiolabeling

*In vivo* labeling of mitochondrially encoded translation products was performed according to the same procedure as previously described [26]. Shortly, cells in logarithmic growth phase (OD_600_ of 1–2) were washed in SGal media without amino acids, after which they were resuspended in SGal with all amino acids lacking methionine. After an incubation time of 5 min at 30°C, ^35^S-labeled methionine was added and aliquots were taken after 5, 10, and 15 min. Unlabeled methionine was added (10 mM) and the temperature increased to 37°C. Aliquots were taken after 30, 60, and 90 min, and proteins were directly extracted with TCA precipitation and analyzed with SDS–PAGE and autoradiography.

### Analysis of mitoribosome interaction via linear sucrose gradients

Isolated mitochondria were suspended in lysis buffer [10 mM Tris–HCl, pH 7.4, 10 mM KOAc, 0.5 mM Mg(OA)_2_, 10 mM EDTA, 5 mM β-mercaptoethanol (β-ME), 1% n-dodecyl-b-d-maltoside (DDM), 1 mM PMSF, 1× complete protease inhibitor (Roche), 0.1 mM spermidine, and 5% glycerol] and lysed for 10 min at 4°C. Lysate was diluted with one volume of lysis buffer without DDM, centrifuged at 16 000 x *g* for 10 min at 4°C, and loaded on a linear sucrose gradient (0.3–1 M sucrose in lysis buffer), which was centrifuged at 164 000 x *g* in a swinging SW60 rotor for 1 h at 4°C. Collected fractions were TCA precipitated, and pelleted proteins were washed twice with acetone and resuspended in sample buffer followed by analysis through SDS–PAGE and western blotting.

### Site-specific photo-crosslinking

Site-specific photo-crosslinking of the conserved chaperone domain of Cbp3 was performed as previously described [27]. Shortly, coding sequences of Cbp3 containing TAG stop codons at selected positions and a C-terminal His7-tag were overexpressed from a pYX142 plasmid (pYX142-Cbp3His7^pBpa^) in strains lacking *CBP3*and/or*MRX4*. These strains also harbored a nuclear-integrated pRS403 plasmid to express the necessary acceptor tRNA^CUA^ (modified *Escherichia coli* tyrosyl tRNA) and aminoacyl-tRNA synthetase [modified *E. coli* tyrosyl-tRNA synthetase (EcYRS-Bpa)] for incorporation of the photoreactive amino acid p-benzoyl-l-phenylalanine (pBpa). Cells grown overnight in SGal-Leu media containing 1.5 mM pBpa were harvested, transferred to a 6-/12-well plate, and exposed to UV light at 350 nm for 60 min. Proteins were extracted through incubation in 0.1 M NaOH (5 min at RT) and lysis in 4% SDS and 100 mM DTT (3 min at 95°C). This was followed by purification of Cbp3His7^pBpa^ and its crosslinking products via Ni-NTA beads and analysis by western blotting, or in case of crosslinking to nascent Cyt*b*, with autoradiography.

### Chemical crosslinking and affinity purification

Mitochondria (5 or 10 mg) from yeast strains expressing protein of interest with a C-terminal ALFA or GFP-ALFA epitope tag were harvested and resuspended in SH buffer (0.6 M sorbitol and 20 mM HEPES, pH 7.4) and incubated for 5 min at 30°C. Membrane permeable chemical crosslinkers m-maleimidobenzoyl-N-hydroxysuccinimide (MBS) or bismaleimidoethane (BMOE) dissolved in dimethyl sulfoxide (DMSO) were added to a final concentration of 200 μM and samples were incubated for 30 min at 30°C, shaking at 600 rpm. DMSO only was added to one sample as non-crosslinked control. Crosslinking was quenched by addition of 100 mM Tris–HCl (pH 8) and 100 mM β-mercaptoethanol followed by incubation for 10 min at 30°C. Mitochondria were pelleted through centrifugation at 25 000 x *g* for 10 min at 4°C and lysed in lysis buffer [20 mM Tris–HCl, pH 7.4, 150 mM KCl, 1% DDM, 1 mM PMSF, 1× Complete protease inhibitor (Roche) and 8 M Urea] for 30 min, tumbling at room temperature. One volume of dilution buffer (20 mM Tris–HCl, pH 7.4, 150 mM KCl, 0.01% DDM) was added and sample was clarified through centrifugation at 16 000 x *g* for 10 min at 4°C. Clarified lysate was added to 40 μl slurry of ALFA selector ST beads (NanoTag Biotechnologies, Göttingen, Germany), incubated for 2 h at room temperature, washed three times with wash buffer A (20 mM Tris–HCl, pH 7.4, 0.1% SDS), three times with wash buffer B (20 mM Tris–HCl, pH 7.4, 6 M Urea), and three times with ABC buffer (50 mM ammonium bicarbonate, pH 8.0) before resuspension in ABC buffer containing 0.5 μg sequencing grade trypsin (Promega). After overnight on-bead tryptic digestion at 37°C, the supernatant was harvested before addition of formic acid to a final concentration of 1%. Peptides were lyophilized for further analysis by mass spectrometry.

### Proximity labeling (Bio-ID)

Proximity labeling via Bio-ID was performed as previously described [18]. In short, yeast strains with protein of interest containing a C-terminal BirA* tag were grown overnight in YPG or YPGal supplemented with 50 μM biotin. The next day, mitochondria were isolated in three biological replicates per strain. Mitochondria (3 mg) were lysed in 1% SDS at 50°C for 5 min, diluted in RIPA buffer [50 mM Tris–HCl, pH 7.5, 150 mM NaCl, 1% NP-40, 1 mM EDTA, 1 mM EGTA, 0.1% SDS, 0.5% sodium deoxycholate, 1× Complete protease inhibitor (Roche) and 1 μl of benzonase (2U, Sigma–Aldrich)], and incubated for 30 min on ice. Lysate was clarified by centrifugation for 10 min at 16 000 x *g* at 4°C and added to pre-equilibrated streptavidin magnetic beads (Thermo Fischer) for 3 h at 4°C. Beads were washed two times with RIPA buffer, two times with TAP lysis buffer (50 mM HEPES, pH 8, 100 mM KCl, 10% glycerol, 2 mM EDTA, and 0.1% NP-40), and three times with ABC buffer (50 mM ammonium bicarbonate, pH 8). Biotinylated proteins were on-bead-digested with 1 μg sequencing grade trypsin (Promega) overnight at 37°C and peptides were lyophilized and analyzed by mass spectrometry.

### Mass spectrometry

For analysis with LC-MS/MS, desalted peptides were injected in an Ultimate 3000 RSLCnano system (Thermo), separated in a 15-cm analytical column (75 mm ID home-packed with ReproSil-Pur C18-AQ 2.4 mm from Dr Maisch) with a 50-min gradient from 5% to 60% acetonitrile in 0.1% formic acid. The effluent from the HPLC was directly electrosprayed into a Qexactive HF (Thermo) operated in data-dependent mode to automatically switch between full scan MS and MS/MS acquisition. Survey full scan MS spectra (from m/z 375–1600) were acquired with resolution *R* = 60 000 at m/z 400 (AGC target of 3 × 106). The 10 most intense peptide ions with charge states between 2 and 5 were sequentially isolated to a target value of 1 × 105 and fragmented at 27% normalized collision energy. Typical mass spectrometric conditions were spray voltage, 1.5 kV; no sheath and auxiliary gas flow; heated capillary temperature, 250°C; ion selection threshold, 33 000 counts. MaxQuant 1.5.2.8 was used to identify proteins and quantify them by iBAQ with the following parameters: Database, UP000002311_559292_Scerevisiae_20171017; MS tol, 10 ppm; MS/MS tol, 0.5 Da; Peptide FDR, 0.1; Protein FDR, 0.01; Min. peptide length, 5; Variable modifications, Oxidation (M); Fixed modifications, Carbamidomethyl (C); Peptides for protein quantitation, razor and unique; Min. peptides, 1; Min. ratio count, 2.

### Selective mitoribosome profiling

Selective mitoribosome profiling was carried out as described [28]. Briefly, yeast were grown in YPGal (1% yeast extract, 2% peptone, 2% galactose), pH 5.0, until an OD_600_ of ∼1. One hundred fifty milliliters of culture was snap chilled over ice prior to formaldehyde crosslinking. The crosslinking was quenched with glycine and washed twice in 40 ml of ice-cold crosslinking wash buffer (50 mM HEPES, pH 7.5, 50 mM NH_4_Cl, 10 mM MgCl_2_). The pellet was then resuspended in 4 ml of crosslinking lysis buffer (50 mM HEPES, pH 7.5, 50 mM NH_4_Cl, 10 mM MgCl_2_, 1.5× Complete EDTA-free protease inhibitor cocktail, 0.5% lauryl maltoside) and dripped into a 50 ml conical filled with liquid nitrogen to form frozen droplets. The frozen cells with lysis buffer were then mechanically lysed using liquid nitrogen-chilled 50 ml canisters for six cycles of 3 min at 15 Hz using a Retsch MM301 cryo-mill. The thawed cell lysate was diluted with 2 ml of fresh crosslinking lysis buffer and treated with 300 units of RNase I (Epicentre) for 30 min in a room temp water bath, swirling halfway to mix. Six hundred units of SUPERase IN RNase inhibitor were added to the lysate to stop RNase digestion. The clarified lysate was layered on top of 8 ml of sucrose cushion buffer (50 mM HEPES, pH 7.5, 50 mM NH_4_Cl, 10 mM MgCl_2_, 1.5× Complete EDTA-free protease inhibitor cocktail, 24% sucrose) and then centrifuged for 4.5 h in a Ti70 (Beckman) rotor at 110 000 x *g* at 4°C.

The ribosome pellet was resuspended in chilled mitoribosome-wash buffer (50 mM HEPES, pH 7.5, 50 mM NH_4_Cl, 10 mM MgCl_2_, 0.1% Triton X-100) overnight at 4°C with shaking. The resuspended ribosome pellet was then added to 50 μl of packed Anti-FLAG M2 Affinity Gel and incubated with end-over-end rotation for 3 h at 4°C. The resin was then washed three times with 1 ml of cold mitoribosome wash buffer. Following the last wash, the resin was resuspended with 1 ml of mitoribosome wash buffer using a cut pipette tip and transferred to a fresh 1.5 ml tube. The wash was removed and the resin was resuspended in 600 μl of mitoribosome wash buffer with 200 ug/ml 3x-FLAG peptide. The resin was then incubated for 1 h at 4°C with rotation. To recover the elution, the resin slurry was passed over a Co-Star SpinX column for 1 min at 16 000 × *g*. The elution was then transferred to a fresh 1.5 ml tube. To reverse the formaldehyde crosslinking, 30 μl of 20% SDS, 32 μl of 100 mM DTT, and 14 μl of 0.5 M EDTA were added to the elution, which was then heated for 45 min at 70°C. The RNA footprints were extracted following reversal of the crosslinking using an equal volume of acid-phenol chloroform. Ribosome footprints were then isolated, and a complementary DNA library was prepared for Illumina short-read sequencing as previously described [29].

### Selective ribosome profiling data analysis

Selective ribosome profiling data analysis was performed as described [28]. Briefly, reads were trimmed, filtered for abundant noncoding RNA, and aligned to the *S. cerevisiae* genome assembly R64 (UCSC: sacCer3) for S288C strain background or assembly ASM216351v1 for W303 strain background. Ribosome A site positions were determined using an offset from the 3′ end of each read, depending on its length: 37, −15; 38, −16; 39, −17; 40, −17; 41, −17. Enrichment was calculated after combining replicates by dividing by the rpm values in a sliding 9-nt window in the corresponding total (Mrps17) dataset. The used scripts are available on Github (https://github.com/churchmanlab/Yeast_selective_mitoRP).

## Results

### Cbs1 and Cbs2 bind to the mitoribosome during early stages of translation

**Figure 1.**
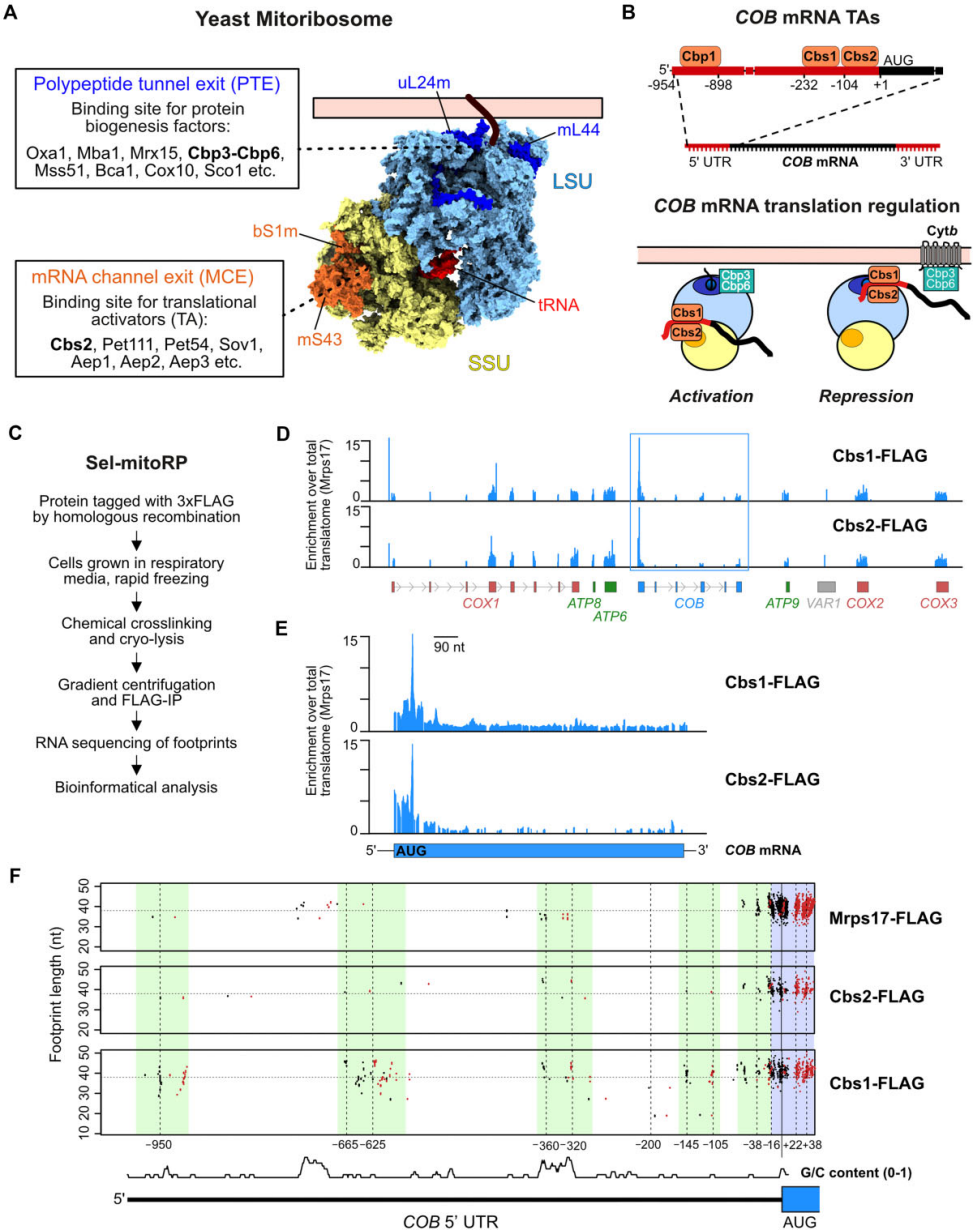
*COB* TAs bind the 5′-UTR of *COB* mRNA and to the MCE on the mitoribosomal SSU. (**A**) Illustration of the yeast mitochondrial ribosome (mitoribosome) highlighting two areas of importance for mitochondrial protein biogenesis. The PTE on the large mitoribosomal subunit (LSU), highlighted with the two proteins uL24m and mL44, is the binding site for several factors involved in protein biogenesis and membrane insertion. The MCE on the small mitoribosomal subunit (SSU), highlighted with the two proteins bS1m and mS43, is the binding site for several proteins involved in activation of translation (TAs). Mitoribosome PDB ID: 5MRC [37]. (**B**) Top: Schematic illustrating the estimated binding regions for the *COB* TA proteins Cbp1, Cbs1, and Cbs2 on the 5′-UTR of the *COB* mRNA. Bottom: The *COB* TAs are proposed to shuttle the *COB* mRNA between a translationally repressed state, bound close to the PTE, and active state where they bind at the MCE for translation initiation. (**C**) General workflow for performing selective mitoribosome profiling (sel-mitoRP) of mitoribosomes crosslinked to and affinity-purified via specific protein factors. (**D**) Genome-wide view of protected mitochondrial RNA footprints after sel-mitoRP of mitoribosomes bound to Cbs1 and Cbs2. Enrichment of TA-specific reads relative to reads from mitoribosomes purified via Mrps17-3xFLAG is presented. (**E**) Sel-mitoRP of Cbs1- and Cbs2-bound mitoribosomes demonstrates an enrichment of footprints only in the 5′ portion of *COB* mRNA and during the start of translation. (**F**) V-plots of protected footprints at specified positions in the 5′-UTR of *COB* mRNA after sel-mitoRP of Cbs1- and Cbs2-bound mitoribosomes compared to non-selective mitoribosomes purified via Mrps17. Regions with marked footprints from the specific TAs and footprints protected by the mitoribosome are highlighted (–16 to +22). GC content: average GC content in 6 nt window (A/T = 0, G/C = 1), scale (0–1) not shown.

The PTE and the MCE on the mitoribosome are two sites particularly important for organizing protein biogenesis and translational regulation, respectively (Fig. 1A). Protein biogenesis factors like membrane insertases and chaperone-like assembly factors bind to the PTE to guide the nascent chain into the membrane and support protein folding [30–33], as also observed in other translation systems [34, 35]. TAs, each specific for one mRNA, bind to the MCE found on the SSU, where a canyon-like structure, formed by mitoribosomal proteins bS1m (Mrp51) and mS43 (Mrp1), serves as their binding platform [18, 28]. Regulation of *COB* mRNA translation entails three TAs, Cbp1, Cbs1 and Cbs2, which were genetically mapped [36] to bind specific portions in the 5′-untranslated region (5′-UTR) of the *COB* mRNA (Fig. 1B). Translational control of *COB* mRNA involves reciprocal localization of either the chaperone Cbp3–Cbp6 or the TAs Cbs1 and Cbs2 in complex with *COB* mRNA to the PTE [17]. We employed selective mitoribosome profiling (sel-mitoRP) [28] to identify the timing of engagement of Cbs1 and Cbs2 with mitochondrial ribosomes (Fig. 1C). For this, RNAse-protected mRNA fragments from mitoribosomes purified by a C-terminally FLAG-tagged uS17m (Mrps17), or through FLAG-tagged variants of Cbs1 or Cbs2, were determined by sequencing. The enrichment of reads found in purifications of Cbs1-FLAG or Cbs2-FLAG relative to whole translatome reads (Mrps17-FLAG) thereby reveals timing of interactions of Cbs1 or Cbs2 with the translating ribosomes. Like in the case of Cbp1 [28], sel-mitoRP showed that ribosomes complexed with Cbs1 and Cbs2 can also be engaged in translation of other mRNAs (Fig. 1D), likely reflecting a state of translational repression of *COB* mRNA due to an activated feedback loop [17]. Importantly, and in line with a specific function during translation initiation and early stages of elongation, we found that Cbs1- and Cbs2-bound mitoribosomes are engaged in translation of the 5′ end of the *COB* ORF, but not at later stages (Fig. 1E). This demonstrates that Cbs1 and Cbs2 play important roles in early stages of *COB* translation but leave the ribosome during later stages of elongation. Likewise, protected fragments of the *COB* mRNA 5′-UTR were observed in Cbs1 sel-mitoRP proximal to the start codon (between –16 and –145), and GC-rich regions were located around –340, –620, and –950 (Fig. 1F). Similar signals were observed for 5′-UTRs of other mitochondrial encoded mRNA [28], suggesting that they represent the TA binding sites on the mRNA.

### Mrx4 is a novel PTE proximity interactor to Cbs2 and Cbp3

**Figure 2.**
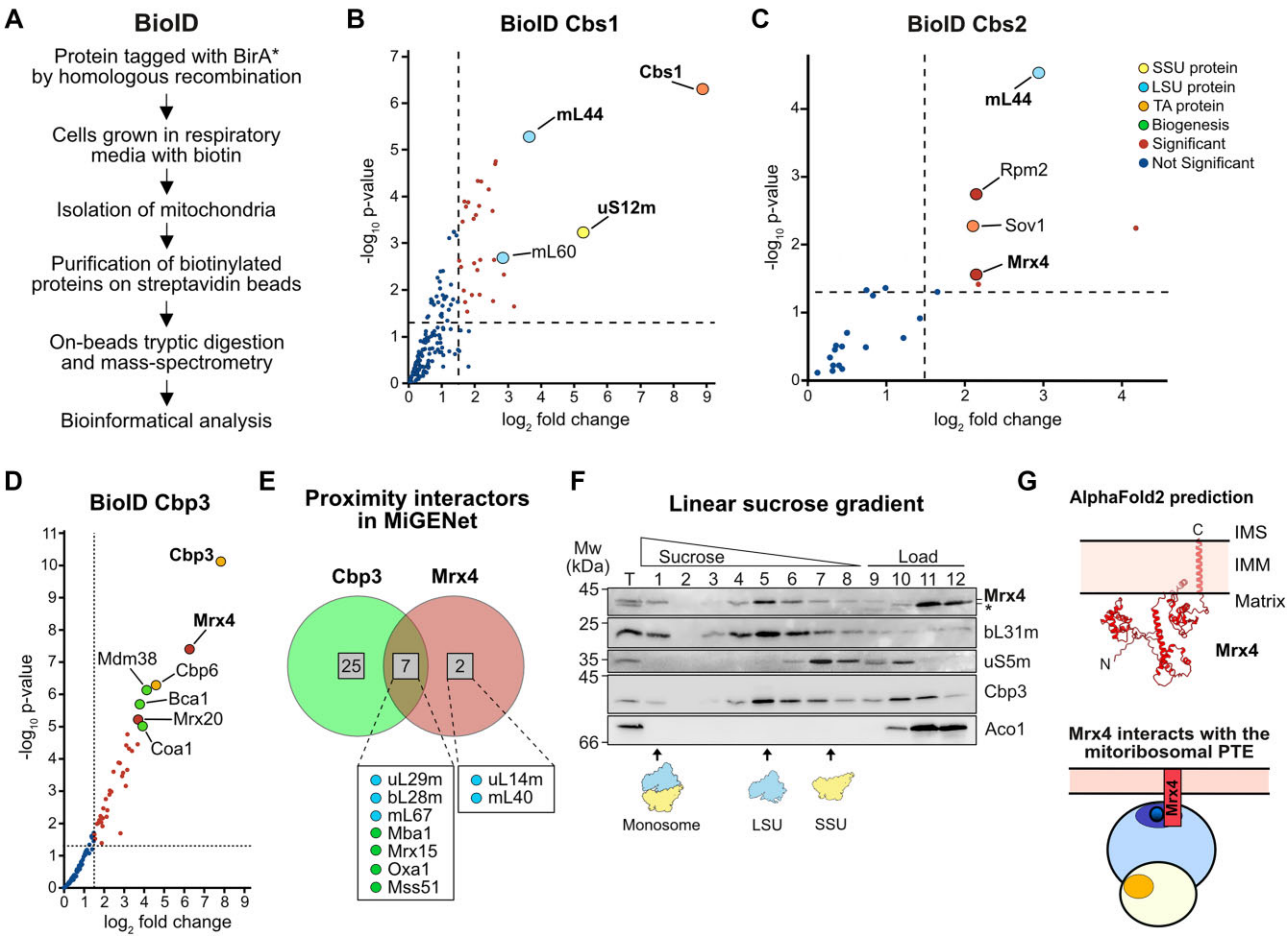
Mrx4 interacts with the PTE, Cbp3–Cbp6, and Cbs2. (**A**) Workflow of proximity labeling (Bio-ID) of mitochondrial proteins using a BirA*-tag genomically introduced to the C-terminus of target proteins. (**B**) Bio-ID of Cbs1 shows proximity to proteins of both the large (LSU) and small (SSU) mitoribosomal subunits. Data presented as log2 fold change compared to a Kgd4-BirA* control with a log_2_ fold change over 1.5 and a log_10_*P* value under 0.5 is considered significant. (**C**) Bio-ID of Cbs2 shows proximity to proteins of the LSU and SSU but also to the *VAR1* TA Sov1 and Mrx4. Data presented as log_2_ fold change compared to a Kgd4-BirA* control. (**D**) Bio-ID of Cbp3 compared as log_2_ fold change compared to the control Kgd4-BirA*. (**E**) First-neighbor analysis of common interactors of Cbp3 and Mrx4 extracted from the proximity interactome network MiGENet [18]. (**F**) Linear sucrose gradient of a mitochondrial lysate shows that Mrx4 interacts quantitatively with the LSU (bL31m) but not with the SSU protein (uS5m) or matrix control Aco1. Cbp3 partially comigrates with the mitoribosome. (**G**) Top: The predicted structure of Mrx4 using AlphaFold2 contains a single transmembrane (TM) helix at the very C-terminal end, with the majority of the protein located in the matrix with a loose fold consisting of three distinct domains. Bottom: Schematic showing Mrx4 interacting with Cbp3–Cbp6 at mitoribosomal PTE.

To further characterize the local environment of Cbs1 and Cbs2 in mitochondria, we utilized Bio-ID analyses. By this, the target proteins are fused to a promiscuous biotin ligase (BirA*), which will mark proteins in proximity to the fusion proteins with biotin, which in turn serves as a handle to purify these proteins by streptavidin and determine their identity by mass spectrometry (Fig. 2A) [18]. Cbs1 was found in proximity to the PTE protein mL44 but also the SSU protein uS12m (Fig. 2B). Proximity mapping of Cbs2 revealed interactions with the LSU protein mL44, Sov1, a TA of *VAR1* mRNA [38] that binds to the MCE of the SSU [18], as well as the uncharacterized, budding yeast-specific protein Mrx4 (Fig. 2C). Furthermore, in a Bio-ID experiment for Cbp3, Mrx4 was one of the most enriched proteins together with Cbp6, the mitoribosome receptor Mdm38 [39], and the complex III assembly factor Bca1 (Fmp25) (Fig. 2D). Mrx4 has previously been identified in proteomic analyses of mitochondrial ribosomes [25] and determined to be a high-confidence proximity interactor of the PTE [18], with a similar pattern as Cbp3 (Fig. 2E).

Likewise, Mrx4 co-migrated with the LSU on a linear sucrose gradient (Fig. 2F), an interaction that was not changed in strains lacking Cbp3 or Cbp1 (which leads to absence of *COB* mRNA) (Supplementary Fig. S1A and B). Similarly, deletion of Mrx4 did not affect the interaction of Cbp3 with the mitoribosome as evidenced by its co-migration with the LSU and the formation of a specific crosslinking product to the PTE protein uL29m (Mrpl4) (Supplementary Fig. S1C–E). We found Mrx4 to localize to the mitochondrial matrix and to be anchored to the inner membrane with a single transmembrane helix (TM) at the C-terminus (Fig. 2G and Supplementary Fig. S1F–I). Interestingly, structure prediction using AlphaFold2 suggested that Mrx4 is largely unstructured with long flexible linkers connecting three more folded domains, which could be important for establishing protein–protein contacts (Fig. 2G).

### Mrx4 mediates the feedback loop necessary for repression of *COB* translation

**Figure 3.**
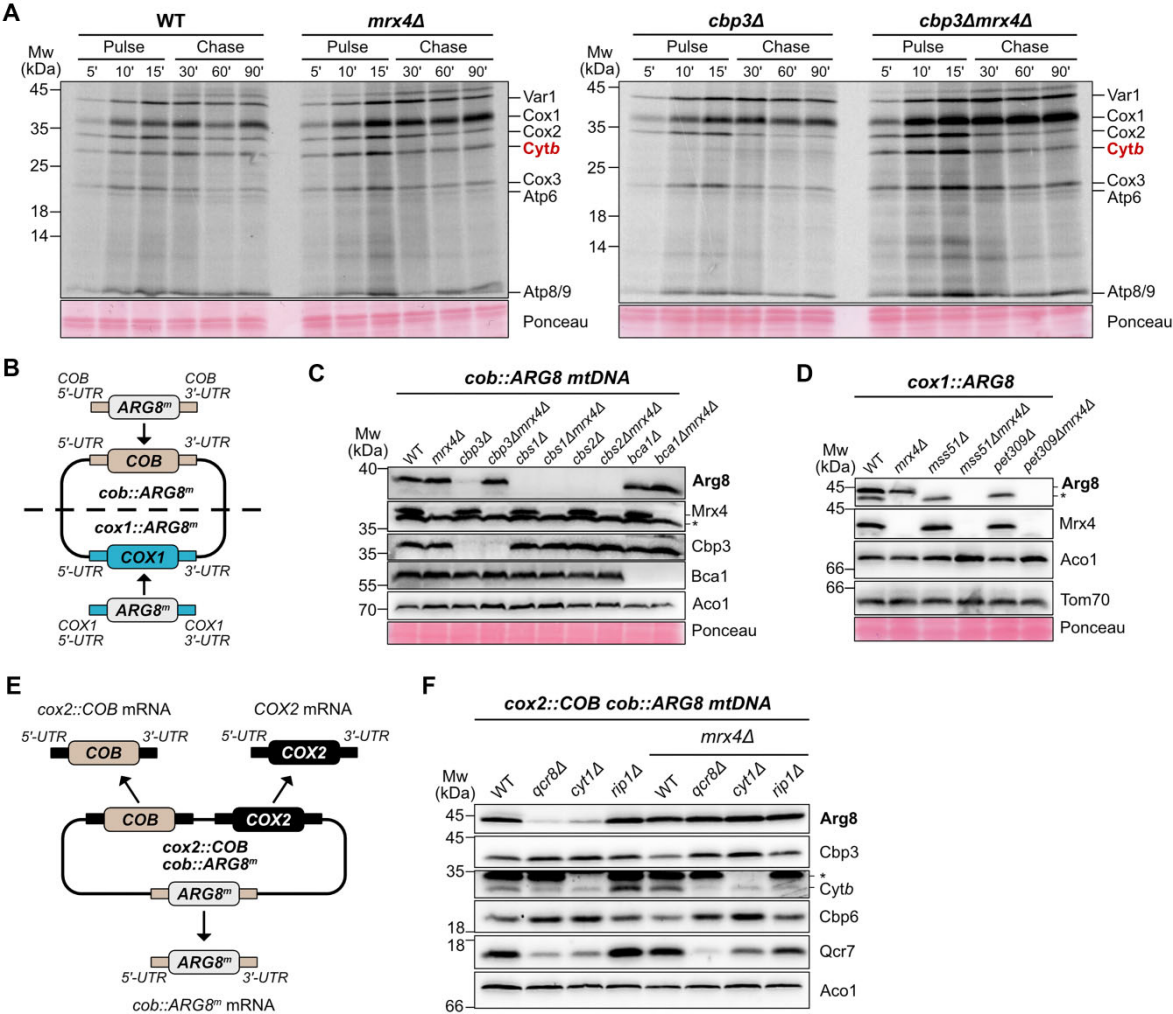
Mrx4 is needed for repression of the *COB* translational feedback loop. (**A**) *In vivo* radiolabeling of indicated strains using ^35^S-methionine. Mitochondrial translation was followed for 15 min (pulse) and the stability of the synthesized proteins was monitored for 90 min (chase). Deletion of *CBP3* leads to a substantial decrease in Cyt*b* synthesis, which is then restored again upon deletion of *MRX4*. (**B**) Schematic showing the mtDNA in the *cob::ARG8* and *cox1::ARG8* reporter strains. *ARG8* has replaced the ORFs of *COX1* and *COB*, with the native 5′- and 3′-UTRs still intact. (**C**,**D**) Steady-state levels of Arg8 and other indicated proteins in *cob:ARG8* and *cox1:ARG8* strains with deletions of *COB* and *COX1* TAs together with deletion of *MRX4*. (**E**) Schematic showing the modifications of mtDNA in the *cox2::COB cob::ARG8* reporter strain. *ARG8* has replaced the ORF of *COB*, with the native 5′- and 3′-UTRs being present, while the native *COB* ORF has been engineered to be expressed under the control of *COX2* 5′- and 3′-UTRs. (**F**) Steady-state levels of Arg8 and other indicated proteins in strains containing the *cox2::COB cob::ARG8* mtDNA together with deletions of *QCR8*, *CYT1*, and *RIP1* in combination with deletion of *MRX4*.

What is the functional relevance of Mrx4 interacting with the PTE? Given that Mrx4 is located in proximity to both Cbs2 and Cbp3, we hypothesized that it might be involved in the regulation of *COB* mRNA translation. Indeed, labeling of mitochondrial translation products with ^35^S-methionine (^35^S-Met) *in vivo* demonstrated that absence of Mrx4 stimulated Cyt*b* synthesis, while the absence of Cbp3 inhibited it (Fig. 3A). Strikingly, in a double mutant lacking both Cbp3 and Mrx4, synthesis rates of Cyt*b* were restored (Fig. 3A). To test directly whether Mrx4 is implicated in translational regulation, we utilized a reporter strain where the *COB* ORF in mtDNA has been replaced with the coding sequence of *ARG8* (Fig. 3B). Accumulation of Arg8, which is produced from an mRNA containing the 5′-UTR of *COB* mRNA, indicates how efficiently this mRNA can be translated, irrespective of whether the produced protein can be assembled into a functional respiratory chain [19]. Deletion of *MRX4* did not decrease levels of Arg8, in contrast to deletion of *CBP3*, which abolished *cob::ARG8* translation (Fig. 3C). Importantly, the decreased accumulation of Arg8 in the *cbp3Δ* strain was completely restored in the double mutant *cbp3Δmrx4Δ*. This effect was specific for Cbp3, as deletion of *MRX4* in cells lacking the other *COB* TAs, Cbs1 and Cbs2, could not restore Arg8 levels (Fig. 3C). These data show that absence of Cbp3 can only repress *COB* translation in the presence of Mrx4, indicating that Cbp3 functions mechanistically different than the TAs Cbs1 and Cbs2. Likewise, deletion of the early complex III assembly factor Bca1 did not affect *cob::ARG8* translation and, subsequently, was not changed upon the deletion of *MRX4*.

Next, we asked whether *MRX4* deletion could also reactivate translation of *COX1* mRNA, another example of a mitochondrially encoded mRNA that is regulated through a feedback loop [22, 40]. Strains lacking the *COX1* TAs Mss51 or Pet309 in the analogous *cox1::ARG8* reporter system (Fig. 3B) showed completely abolished accumulation of Arg8 (Fig. 3D). Importantly, additional deletion of *MRX4* in the *mss51Δ* or *pet309Δ* strains did not restore Arg8 levels, revealing that Mrx4 plays a dedicated, direct role in Cbp3–Cbp6-dependent translation of *COB* mRNA.

Cbp3–Cbp6 shuttles between binding to newly synthesized Cyt*b* or to the mitoribosome, which leads to a new round of *COB* translation [20]. Absence of nuclear-encoded, imported complex III subunits, like Qcr7, Qcr8, or cytochrome *c*1 (Cyt1), stalls complex III assembly and sequesters Cbp3–Cbp6 in early assembly intermediates. This translational repression can be clearly demonstrated in a strain where the native *COB* ORF is replaced by *ARG8*, while a second engineered *COB* ORF is expressed from an mRNA flanked by the *COX2* UTRs (Fig. 3E) [20]. This results in the constitutive expression of *COB* by the *COX2* TAs, while *ARG8* serves as a reporter for translational efficiency of the *cob::ARG8* mRNA. Analysis with western blot (Fig. 3F) showed markedly decreased levels of Arg8 in cells lacking Qcr8 and Cyt1, while absence of the late-assembling Rip1 did not change Arg8 levels. Intriguingly, deletion of *MRX4* in these strains restored Arg8 synthesis to wild-type (WT) levels, demonstrating that absence of Mrx4 disrupts the *COB* translational feedback loop.

### Mrx4 interacts with the Cyt*b*-binding site of Cbp3

**Figure 4.**
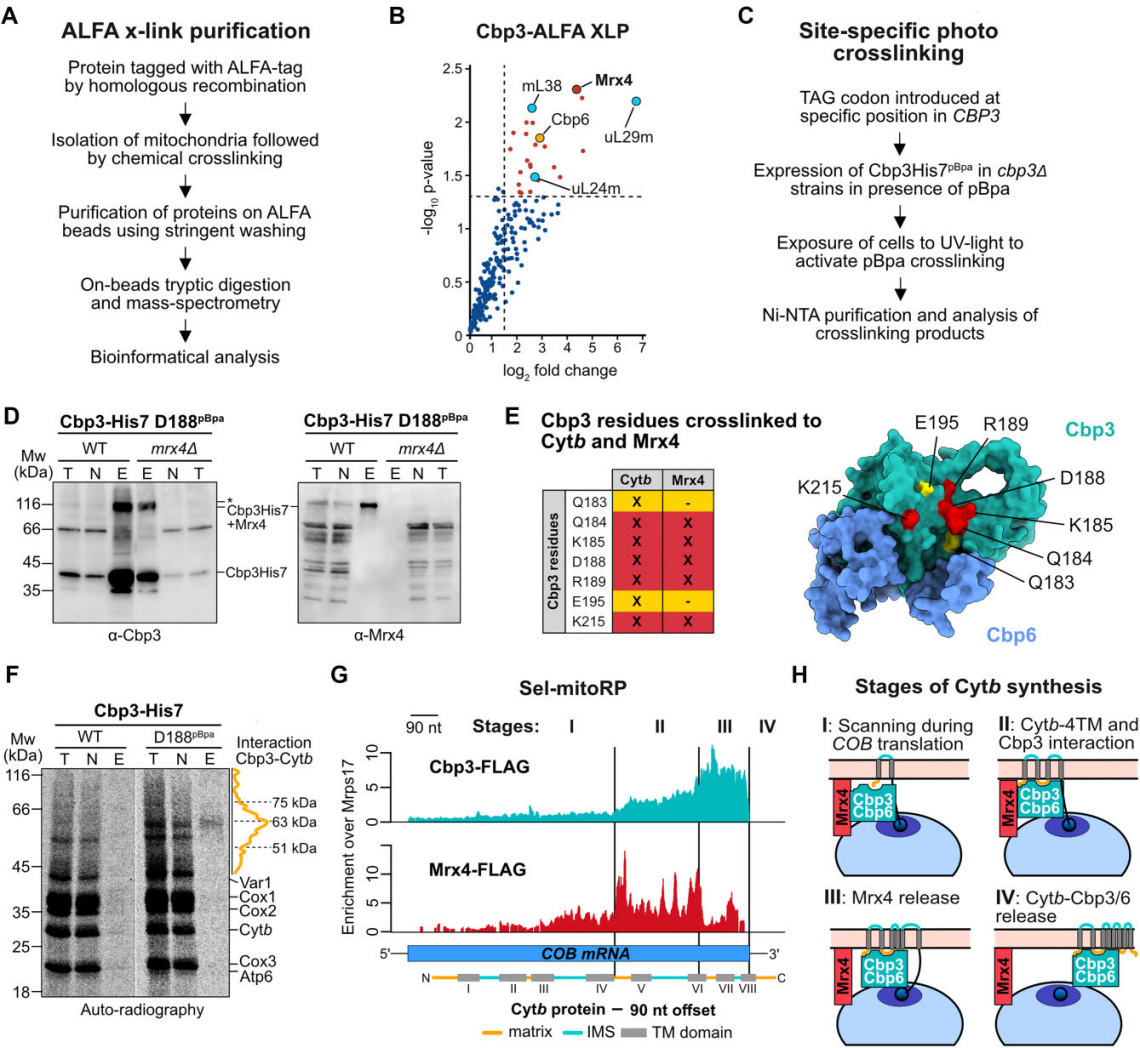
Mrx4 coordinates activating and repressive signals at the PTE. (**A**) Workflow for the stringent purification of proteins with a genomically integrated ALFA-tag following chemical crosslinking and with stringent washing. Samples were subsequently analyzed via on-bead tryptic digestion and mass spectrometry. (**B**) ALFA crosslinking purification (ALFA-XLP) of Cbp3-ALFA followed by analysis with mass spectrometry. Data presented as log_2_ fold change compared to a Cbp3-ALFA control without crosslinking and with a threshold set to 1.5 (*n* = 3 for both conditions). (**C**) Scheme of the experimental approach for site-specific photo-crosslinking of His7-tagged Cbp3 by incorporation of the photoreactive amino acid p-benzoyl-l-phenylalanine (pBpa) at specific positions. Crosslinking was followed by Ni-NTA purification and analysis via western blotting. (**D**) Site-specific photo-crosslinking and purification of Cbp3-His7 with pBpa incorporated at residue D188 in the presence or absence of Mrx4. Decoration with antibodies against Cbp3 and Mrx4 shows that a crosslinking product that contains Mrx4 is co-purified with Cbp3-His7 at this specific position. *Signal at a similar position as the Mrx4-Cbp3 crosslinking product in the elution fraction decorated with the Cbp3 antibody likely reflects another crosslinking product of Cbp3. T = total, N = non-bound, E = elution. (**E**) Left: Table showing Cbp3 residues that previously have been photo-crosslinked to Cyt*b* [27] and now also to Mrx4. Right: Mapping of these residues on the AlphaFold2-predicted structure of Cbp3–Cbp6 (Cbp3 in green and Cbp6 in blue) points to a shared binding site of Cyt*b* and Mrx4 on the conserved chaperone domain of Cbp3. (**F**) Densitometric quantification of the smeary nascent Cyt*b* crosslinked to Cbp3 after *in organello* labeling with ^35^S-methionine followed by site-specific photo-crosslinking and purification of residues Q184, D188 (visualized), and K215. T: total, N: non-bound, E: elution. (**G**) Selective mitoribosome profiling (sel-mitoRP) of Cbp3- and Mrx4-bound mitoribosomes demonstrates enrichment during the later stages of *COB* translation. Data presented as enrichment over mitoribosomes purified via Mrps17-3xFLAG. Cyt*b* is depicted with a 90 nt offset to represent emergence through the polypeptide tunnel. (**H**) Illustration of the four suggested stages of Cyt*b* synthesis with regard to interactions of Cbp3–Cbp6 (green) with Mrx4 (red) and the Cyt*b* nascent chain.

To investigate whether Cbp3 directly interacts with Mrx4, we used chemical crosslinking in intact mitochondria containing ALFA-tagged protein followed by purification and analysis with mass spectrometry (ALFA-XLP) (Fig. 4A). This confirmed the established interactions of Cbp3 with its partner protein Cbp6 and with the PTE proteins uL29m (Mrpl4) and uL24m (Mrpl40) [19], but also revealed a direct binding of Cbp3 to Mrx4 (Fig. 4B).

To further characterize the interaction between Cbp3 and Mrx4, we utilized site-specific photocrosslinking using the photoreactive amino acid p-benzoyl-l-phenylalanine (pBpa) incorporated at specific sites in Cbp3 [27]. After exposure to UV light, pBpa will crosslink to neighboring residues yielding a covalent protein–protein crosslinking product that can be analyzed through purification and western blotting (Fig. 4C). Using this approach, we have previously mapped the substrate binding site of Cbp3 [27], which binds to Cyt*b* and is formed by a V-shaped helix-turn-helix motif [41]. Strikingly, the Cbp3 substrate binding site also formed specific crosslinks with Mrx4 at five residues (D188pBpa, Q184pBpa, K185pBpa, R189pBpa, and K215pBpa) (Fig. 4D and E and Supplementary Fig. S2A). Therefore, Mrx4 binds directly to the substrate-binding site of Cbp3–Cbp6, an interaction that should be dissolved by binding of nascent Cyt*b* to Cbp3.

To reveal an interaction between nascent Cyt*b* and Cbp3, we used site-specific photocrosslinking to the substrate binding site in combination with labeling of mitochondrial translation products with ^35^S-Met. Purification of Cbp3 after crosslinking resulted in a specific signal of full-length Cyt*b* and a lower-molecular-weight smear reflecting nascent Cyt*b* interacting with Cbp3 (Fig. 4F and [27]). To determine the exact timing of the interactions between Cbp3 and Mrx4 with the mitoribosome during Cyt*b* synthesis, we performed sel-mitoRP on cells expressing Flag-tagged Cbp3 or Mrx4, respectively (Fig. 4G). While only low-intensity signals were recorded for Cbp3 sel-mitoRP during early stages of *COB* translation, increased interactions were observed at a stage where the first four TMs of Cyt*b* have emerged from the ribosome. This signal was even further intensified at late stages of Cyt*b* synthesis. Mrx4 sel-mitoRP revealed a similar trend, but the signal of Mrx4 strongly declined upon emergence of the sixth TM of Cyt*b*. Taken together, these data reveal four different stages during Cyt*b* synthesis (Fig. 4H): a first step (I), where Cbp3–Cbp6 bound to Mrx4 scans the PTE for emergence of the nascent Cyt*b*; a second step (II), where Cbp3–Cbp6 establishes an interaction with the portion of Cyt*b* encompassing the first four TMs. In the next step (III), the interaction between Mrx4 and Cbp3 is dissolved around emergence of the sixth TM segment. Finally (IV), the complex containing fully synthesized Cyt*b* and Cbp3–Cbp6 leaves the ribosome upon completion of translation.

### Mrx4 orchestrates the *COB* translational feedback loop at the PTE

**Figure 5.**
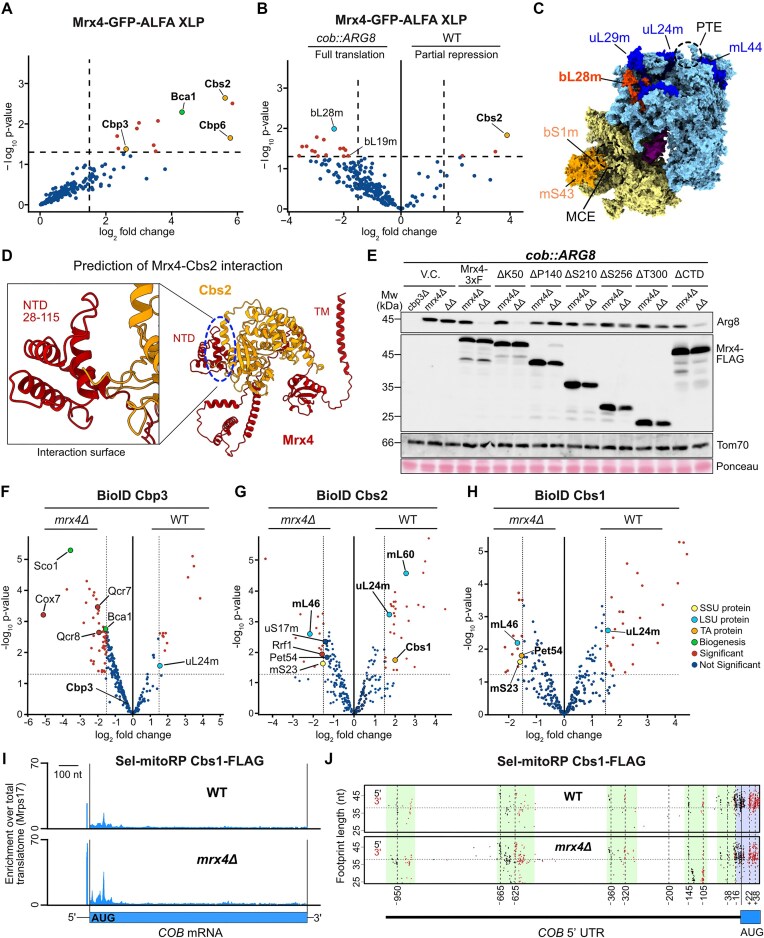
*COB* TAs shuttle the mRNA between the LSU and SSU. (**A**) Purification of Mrx4-GFP-ALFA from isolated mitochondria treated with the chemical crosslinker MBS (ALFA-XLP). Data presented as log_2_ fold change compared to a control treated with DMSO and with a threshold set to 1.5 (*n* = 3 for both conditions). (**B**) ALFA XLP of Mrx4-GFP-ALFA in WT compared to the *cob::ARG8* genetic background, reflecting partial repression and full activation of *COB* translation, respectively. (**C**) Illustration of the location of mitoribosomal proteins on the structure. (**D**) AlphaFold2 prediction of Mrx4 (red) in complex with Cbs2 (orange). The main interaction is predicted to occur on an NTD of Mrx4 spanning residues 52–115. TM: transmembrane helix. NTD: N-terminal domain. (**E**) Steady-state levels of Arg8 in *cob::ARG8 mrx4Δ* and *cbp3Δmrx4Δ* (ΔΔ) strains expressing truncated versions of Mrx4-3xFLAG from a pYX132 plasmid to test repressor function of Mrx4. To allow import of the N-terminally truncated variants into mitochondria, the authentic N-terminus was replaced by an Oxa1 mitochondrial targeting signal at indicated residues and for the CTD mutant (ΔCTD), the sequence for a 3xFLAG tag followed by a stop codon was inserted before the TM helix (Supplementary Fig. S2B and C). V.C. = empty pYX132 vector control. Proximity labeling of (**F**) Cbp3-BirA*, (**G**) Cbs2-BirA*, and (**H**) Cbs1-BirA* in the presence (WT) or absence of Mrx4 (*mrx4Δ*) with data presented as significant with a log_2_ fold change over 1.5 and a log_10_*P*-value under 0.5. (**I**) Selective mitoribosome profiling (sel-mitoRP) of Cbs1-bound mitoribosomes shows enrichment during initiation of *COB* translation, which is markedly increased (2.5×) in cells lacking *MRX4*. Both sets of data are presented as enrichment over mitoribosomes purified via Mrps17-3xFLAG in WT or *mrx4Δ* strain backgrounds, respectively. (**J**) V-plot of protected footprints at specified positions in the 5′-UTR of *COB* mRNA after sel-mitoRP of Cbs1-bound mitoribosomes in WT and *mrx4Δ* strains. The five main regions of footprints all show more reads in the strain lacking Mrx4, indicating a higher occupancy of *COB* TAs. The region with footprints protected by the mitoribosome is highlighted in blue.

Having identified the timing of Cytb–Cbp3–Cbp6 interaction, we next aimed to unravel how the absence of Mrx4 can disrupt the regulation of *COB* translation. ALFA-XLP demonstrated that Mrx4 binds to Cbp3–Cbp6, the assembly factor Bca1, and, intriguingly, also to the *COB* TA Cbs2 (Fig. 5A). Because binding of Cbs2 to the PTE coincides with repression of Cyt*b* synthesis [17], while binding of Cbp3–Cbp6 to the PTE activates translation [20], we hypothesized that the interaction of these factors with Mrx4 might be central to orchestrating the feedback loop by reciprocal interactions.

To test this, we compared interaction patterns of Mrx4 in cells with fully active translation of *COB* mRNA (using a strain with the *cob:ARG8* mtDNA lacking Cyt*b*, hence without sequestration of Cbp3–Cbp6 in assembly intermediates) with the WT scenario, where *COB* translation is substantially repressed due to feedback inhibition [21]. As predicted, full translation in the strain carrying *cob:ARG8* impaired the contact between Mrx4 and Cbs2, which was clearly enriched in WT mitochondria (Fig. 5B). Moreover, Mrx4 contacted the LSU protein bL28m (Mrpl28), which localizes on the ribosome between the PTE and MCE, during full translation, suggesting that Mrx4 switches localization (and presumably changes conformation) in a *COB*-translation-dependent manner. While under WT conditions, Bio-ID revealed that Mrx4 is primarily bound to the PTE in vicinity of mL44 and Cbs2 (Fig. 2C); the contact to Cbs2 is decreased and new contacts with bL28m are established during full *COB* translation (Fig. 5C).

Structure prediction using AlphaFold2 suggested that Mrx4 interacts with Cbs2 with a small globular domain spanning residues 30–115 (Fig. 5D), a contact presumably representing the repressed state of *COB* translation. To test whether repression indeed depends on this predicted interaction, we expressed truncated variants of Mrx4 in cells harboring the *cob::ARG8* mtDNA and lacking both Cbp3 and Mrx4 (Fig. 3B), where the *cob::ARG8* mRNA was fully translated (Fig. 3C). Specifically, we identified folded regions in the predicted structure of Mrx4 and truncated the protein between these domains (Supplementary Fig. S2B–E). Expression of full-length Mrx4 or a mutant lacking the first 50 amino acids of the mature N-terminus restored repression, while expression of constructs lacking 140 or more residues of the N-terminus could not repress Arg8 synthesis (Fig. 5E). These data indicate that Mrx4 contacts Cbs2 with a domain located between residues 50 and 140 to mediate translational repression of the *COB* mRNA.

Having identified Mrx4 as the key component of this translational feedback loop, we next sought to understand the localization dynamics of the *COB* TAs and assembly factor with and without Mrx4. Using BioID, we found that Cbp3 binding to the PTE (as evidenced by its proximity to uL24m) was enhanced in the presence of Mrx4, while Cbp3 increased proximity to respiratory chain assembly factors and early assembling subunits in the absence of Mrx4 (Fig. 5F). Comparing the Cbs1 and Cbs2 proximity interactomes revealed differences in WT and *mrx4*Δ cells, reflecting the switch from partial repression to full translation of *COB* mRNA (Fig. 5G). In WT cells, these *COB* TAs were located close to the PTE (as evidenced by uL24m), revealing its interactions during the repressed state of *COB* mRNA translation. However, in the absence of Mrx4, they changed sub-ribosomal localization and were found at the SSU and the MCE (Fig. 5G and H). Consequently, sel-mitoRP of Cbs1 in the absence of Mrx4 revealed substantially increased reads in the 5′ end of the *COB* mRNA (Fig. 5I), concomitant with increased footprints of protected fragments in the 5′-UTR (Fig. 5J), indicating increased translation initiation upon disruption of the translational feedback loop.

Together, these data reveal that Mrx4 orchestrates a spatial reorganization of both *COB* TAs and assembly factors, maintaining TAs in proximity to the PTE when translation needs to be restrained, while also ensuring proper PTE positioning of assembly factors for efficient complex III assembly. In the absence of Mrx4, these factors undergo distinct relocalizations—TAs shift to sites of translation initiation leading to enhanced Cyt*b* synthesis, while assembly factors show increased associations with other respiratory chain assembly components, likely reflecting their redirected activities when the feedback loop is disrupted. These findings establish Mrx4 as the molecular switch that controls this translational feedback loop through its ability to dynamically regulate the spatial distribution of both TAs and assembly factors at the mitoribosome.

## Discussion

Mitochondrial translation is highly specialized to produce a handful of mostly very hydrophobic proteins. The small number of protein-coding genes in mtDNA facilitated the establishment of mechanisms that allow for a tailored biogenesis of its translation products, including highly specialized mechanisms for translational regulation. The coordination of mitochondrial and nuclear gene expression is a key challenge during the biogenesis of the OXPHOS system, which is solved in the case of yeast Cyt*b* through a translational feedback loop targeting translation initiation of the *COB* mRNA. Here, we have identified how Mrx4, a ligand of the mitoribosomal PTE, acts as a molecular switch in this process. Mrx4 provides a binding site for *COB* mRNA complexed with two TAs, which is necessary to repress its translation. The presence of the assembly factor Cbp3–Cbp6 at the PTE and interaction with Mrx4 activates the switch, triggering the release of the *COB* TA complex, which can then interact with MCE for translation initiation. Through this mechanism, the presence of Cbp3–Cbp6 at the PTE can be sensed and integrated into a signal for enhanced Cyt*b* synthesis.

Translational repression in the cytosol works by various strategies, including sequestering mRNAs into higher-order structures to exclude these mRNAs from translation initiation [42, 43], by inhibiting key initiation factors by direct interactions or through posttranslational modifications [44, 45], or through mechanisms of RNA interference [46]. Organellar protein synthesis depends on and reacts to nuclear gene expression [1, 2], but also maintains mechanisms for translational regulation [7, 13, 47]. For this, endosymbiotic organelles like mitochondria and chloroplasts contain specific RNA-binding proteins [48], [49], which often contain pentatricopeptide repeat (PPR) motifs to recognize specific mRNAs. Many of these RNA-binding proteins in yeast thereby interact with one specific mRNA, often in 5′-UTRs, and are necessary to activate translation of this mRNA. Previous work has shown that these TAs bind to the MCE [18, 28] to help align the start codon into the ribosomal P-site, thereby replacing the missing Shine–Dalgarno sequence as molecular guides [28]. Protected fragments of the 5′-UTR of *COB* mRNA revealed in this work suggest that the TAs bind this mRNA in a folded state that spans large portions of the mRNA, similar to the case of the *ATP9*–Aep1–Aep2–Atp25C complex [28]. Specifically, the portion around 100 nucleotides relative to the start codon is likely important for translation initiation, as this part should determine start codon alignment. Previous genetic mapping [36] revealed that this region of the 5′-UTR is bound by Cbs2, which in turn binds to the MCE. Hence, it is conceivable that Cbs2 plays a dedicated role for aligning the *COB* mRNA into the mRNA channel, while Cbs1 and Cbp1 could impose or stabilize a specific fold of the 5′-UTR, which would require a certain flexibility to allow for the observed translocations of the TA complex from the MCE to the PTE and vice versa.

**Figure 6.**
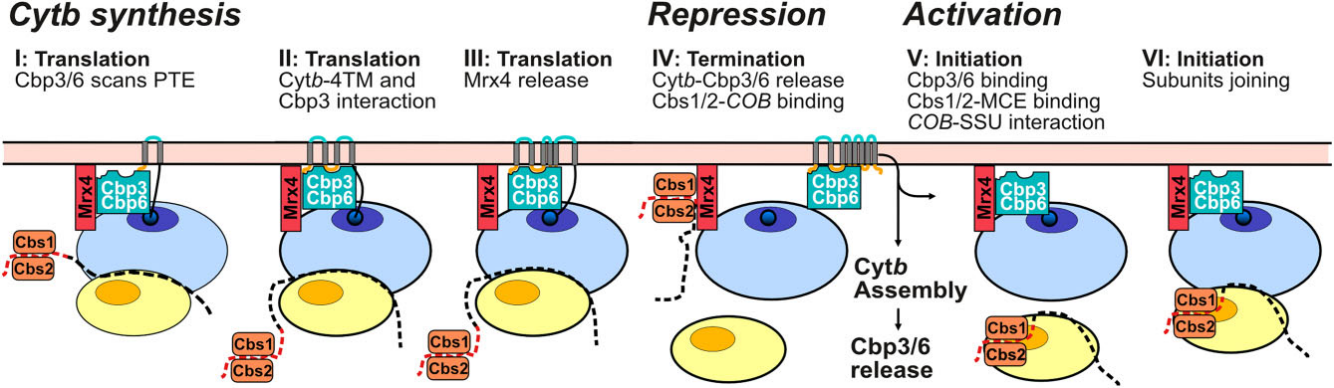
Orchestration of Cyt*b* biogenesis and translation control at the mitoribosomal polypeptide tunnel exit. A model of the translational feedback loop and dynamics of protein biogenesis of the mitochondrial encoded Cyt*b*. Cyt*b* biogenesis relies on distinct steps of protein interactions during *COB* translation to ensure that newly synthesized Cyt*b* can be bound to its dedicated chaperone Cbp3–Cbp6. Once Cyt*b* is fully synthesized, it leaves the mitoribosome in a complex with Cbp3–Cbp6. Through this, the PTE ligand Mrx4 is available to bind the *COB* TA Cbs2, thereby sequestering the *COB* TA complex at the PTE. Release of Cbp3–Cbp6 from Cyt*b* during assembly allows to activate a new round of *COB* translation. To this end, Cbp3–Cbp6 competes off the *COB* TA complex from Mrx4. The thereby released *COB* TA complex can now interact with the MCE at the SSU for translation initiation.

The PTE is the site of early protein biogenesis. Upon emergence from the tunnel exit, early folding events and protein maturation occur that are often mediated by specific proteins, including chaperones, membrane insertases, and processing enzymes [35, 50]. These early biogenesis factors interact with ribosomal proteins decorating the surface surrounding the PTE. Here, we have identified Mrx4 as a novel ligand of the mitoribosomal PTE that does not play a role in early protein biogenesis but is necessary for translational regulation of a single mRNA. To this end, Mrx4 interacts reciprocally with either the *COB*-TA complex or the Cyt*b*-specific chaperone Cbp3–Cbp6. Interestingly, Mrx4’s crosslinking partners were altered during fully active translation, which would be in line with a conformational change upon loss of interaction with *COB*-TA and binding to Cbp3–Cbp6. Strikingly, both Mrx4 and Cyt*b* engage with the substrate binding site of Cbp3. Hence, the interaction between Cbp3–Cbp6 and Cyt*b* apparently competes off Cbp3–Cbp6 from Mrx4, revealing the time point when the translational switch is activated. Accordingly, a plausible model for *COB* translational control (Fig. 6) is that Mrx4 binds the *COB* TAs. Once Cbp3–Cbp6 detaches from Cyt*b*, the interaction of this assembly factor with Mrx4 releases the *COB* TAs. The TAs, bound to *COB* mRNA, are then free to localize to the MCE, enabling translation initiation. Early during elongation, the TAs leave the MCE and the mitoribosome. Meanwhile, Mrx4-bound Cbp3–Cbp6 scans the nascent Cyt*b* at the PTE. Upon emergence of the loop after the fourth TM segment from the ribosome, Cbp3–Cbp6 binds the nascent Cyt*b* [41]. Subsequently, Cbp3–Cbp6 is released from Mrx4 (when the sixth TM segment emerged from the tunnel), upon which Mrx4 is available to “reload” through interacting with the *COB*-TA complex and inhibit further translation initiation on *COB*. Finally, a complex containing full-length Cyt*b* and Cbp3–Cbp6 detaches from the mitoribosome to mediate Cyt*b* maturation and assembly. Once Cyt*b* is fully hemylated and interacts with the first nuclear-encoded subunits [21], Cbp3–Cbp6 is released from Cyt*b* to activate a new round of *COB* translation.

In addition to *COB* mRNA, *VAR1*, *COX1*, and *ATP6*, coding for key subunits of the small mitoribosomal subunit, cytochrome *c* oxidase, or ATP synthase, respectively, are subject to translational regulation in yeast mitochondria [22, 38, 40, 51]. In these cases, sequestration of the translation products in assembly intermediates represses new rounds of translation, but how this is relayed to translation is yet unknown. Cox14 is a cytochrome *c* oxidase assembly factor that stabilizes the Cox1-containing assembly intermediate important for translational repression [40, 52, 53]. In its absence, this assembly intermediate cannot form efficiently, and hence sequestration of the regulating TA, Mss51, in the intermediate is less efficient, leading to a restoration of *COX1* translation. The identification of Sov1 as a proximity interactor of Cbs2 could suggest that both TAs are bound simultaneously to the ribosome, but it is yet unclear whether this occurs during translation of *COB* or of *VAR1* mRNA and whether this would allow coordinate expression of both transcripts. Interestingly, the protein Smt1 (Mrx5) was recently identified as a repressor of Atp6 and Atp8 synthesis [54] that binds the bicistronic *ATP8*/*ATP6* mRNA. How Smt1 mediates this repression is yet unknown. However, given that Smt1 interacts with mitoribosomes [25], it is possible that it operates through a similar mechanism as Mrx4. It will be an exciting task for future research to identify these molecular mechanisms that will likely show compelling variations of translational regulation in mitochondria.

## Supplementary Material

gkaf634_Supplemental_Files

## Data Availability

Raw and processed sequencing 645 data generated in this study were deposited in the GEO database under the accession number 646 GSE283176. Total mitoribosome footprints (Mrps17) for S288C strains are available under 647 accession number GSE282943. Original data from western blotting and autoradiography analyses are deposited at Mendeley (doi: 10.17632/9kkbp885cs.1). Spreadsheets containing original data and analyses for mass spectrometry are included as Supplementary Tables S2–S5. The used scripts are available on Github (https://github.com/churchmanlab/Yeast_selective_mitoRP) and Zenodo (https://doi.org/10.5281/zenodo.15679476).
