# Supplementary material for: A molecular switch at the yeast mitoribosomal tunnel exit controls cytochrome b synthesis
Source: Nucleic Acids Res. 2025 Jul 10;53(13):gkaf634. doi: 10.1093/nar/gkaf634 (PMC12242767; doi:10.1093/nar/gkaf634)
Supplement: gkaf634_Supplemental_Files [file gkaf634_supplemental_files.zip › Supplemental figures+legends.pdf]

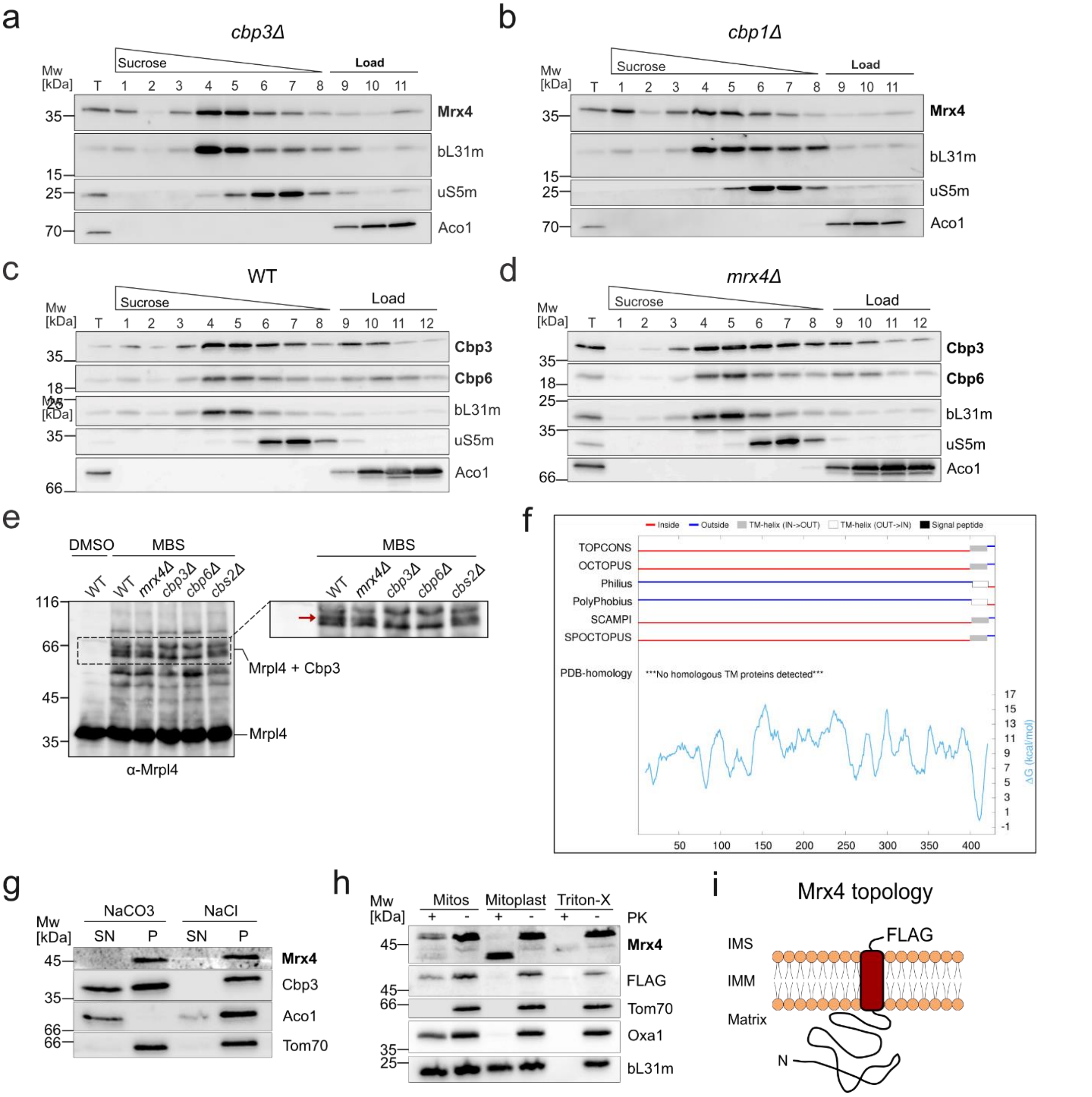

Supplemental figure 1: Sucrose density gradients using mitochondria, where **(a)** *CBP3* or **(b)** *CBP1* have been deleted, show no effect on Mrx4 co-migration with the mitoribosome. Sucrose density gradients demonstrating that co-migration of Cbp3-Cbp6 with the mitoribosome does not change in the **(c)** presence or **(d)** absence of Mrx4. **(e)** *In situ* chemical crosslinking reveals binding of Cbp3 to the PTE in intact mitochondria in the absence of Mrx4. Mitochondria isolated from the indicated strains where exposed to the chemical crosslinker MBS. After quenching, proteins were separated via SDS-PAGE and analyzed with Western blotting against Mrpl4 (uL29m). A specific crosslinking product just below 66 kDa is formed that is absent in strains lacking Cbp3 or Cbp6, but not when Cbs2 or Mrx4 are deleted. **(f)** Topology prediction using *TOPCONS* for Mrx4 (YPL168W) predicts it to be an integral membrane protein with a single transmembrane helix close to the C-terminal end. **(g)** Mitochondria where exposed to alkaline treatment ($NaCO_3$) or high salt (NaCl) and separated into a membrane fraction (P) and a soluble fraction (SN). Mrx4

behaved as the membrane protein Tom70. **(h)** Mitochondria in isotonic media (Mitos), in hypotonic media (Mitoplast) or dissolved in detergent (Triton-X) were exposed to proteinase K (PK). Mrx4, carrying a C-terminal FLAG-tag, was protected from PK in intact mitochondria and cleaved into an N-terminal fragment in mitoplasts, establishing the topology depicted in **(i)**.

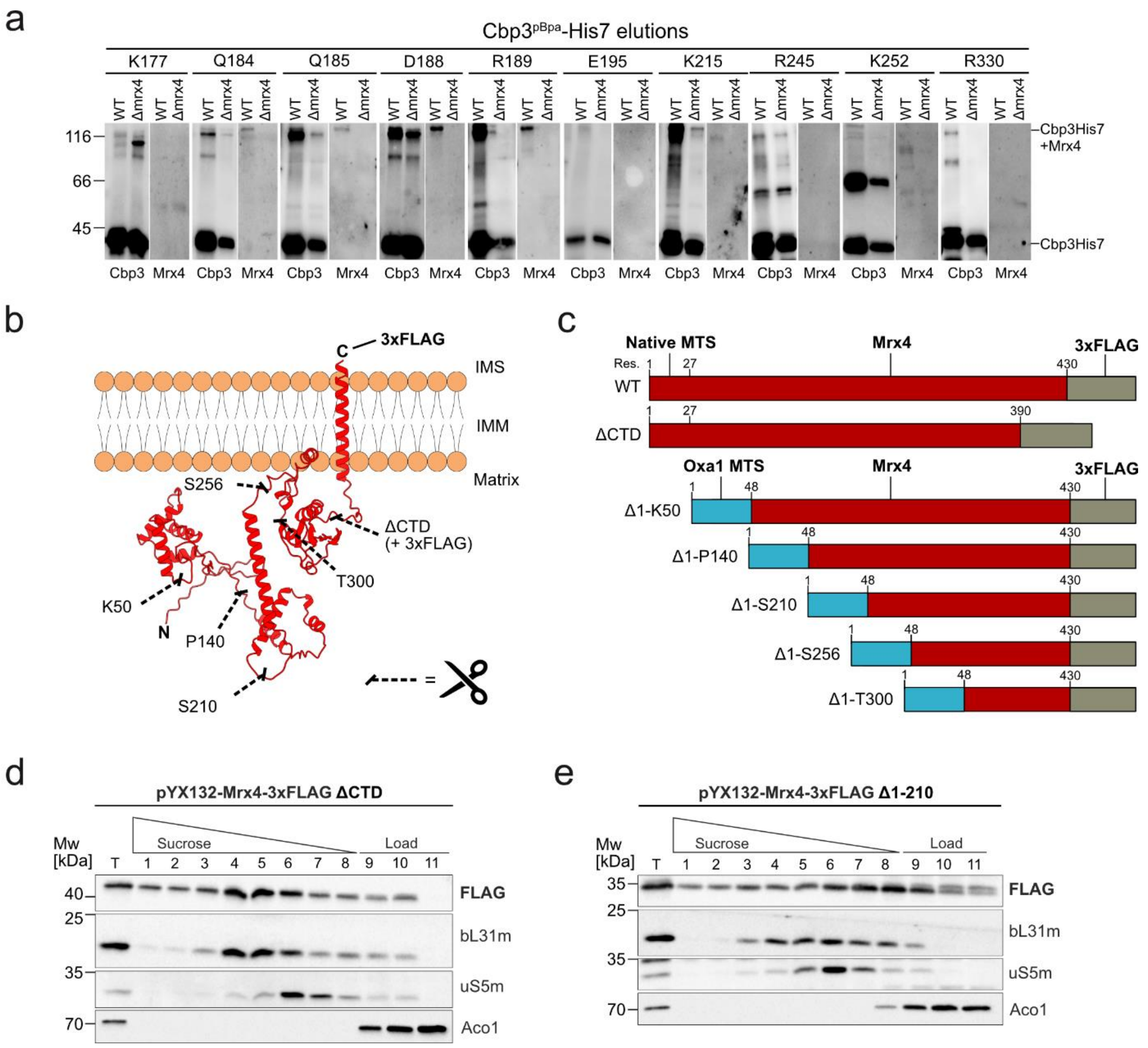

Supplemental figure 2: **(a)** Site-specific photo-crosslinking and purification of Cbp3-His7 with the photoreactive amino acid p-benzoyl-L-phenylalanine (pBpa) incorporated at indicated positions in either wild-type (WT) or a strain lacking *MRX4*. Elution fractions decorated with antibodies against Cbp3 and Mrx4 shows co-purification of a crosslinking product around 116 kDa, at position Q184, Q185, D188, R189 and K215, which is not present in absence of Mrx4. Other crosslinking products of similar size can also be seen when decorating with the Cbp3 antibody. **(b)** Structure prediction of Mrx4 using *AlphaFold2* shows a single transmembrane helix at the C-terminus and most of the protein folded into small domains separated by large flexible linkers. The predicted domains were sequentially removed at the indicated positions (dashed lines) to generate truncated Mrx4 variants. **(c)** Schematic depicting the different Mrx4 truncation constructs

used in Fig 5e. The *MRX4* coding sequence (red) followed by a C-terminal 3xFLAG epitope tag was first cloned into pYX132. For the Mrx4 C-terminal truncation (ΔCTD), the 3xFLAG sequence was instead added after residue 390 to remove the predicted single TM helix. For Mrx4 N-terminal truncations, the Oxa1 mitochondrial targeting sequence (MTS) was cloned into the indicated positions (K50, P140, S210, S256 and T300) to facilitate proper import of the truncated variants. Sucrose density gradients of mitochondrial lysates expressing either the **(d)** Mrx4 ΔCTD or **(e)** Δ210 truncation variants show that the C-terminal TM helix is not necessary for Mrx4 interaction with the mitoribosomal large subunit, while this interaction is largely lost in the Δ210 N-terminal variant.
