# Supplementary material for: A molecular switch at the yeast mitoribosomal tunnel exit controls cytochrome b synthesis
Source: Nucleic Acids Res. 2025 Jul 10;53(13):gkaf634. doi: 10.1093/nar/gkaf634 (PMC12242767; doi:10.1093/nar/gkaf634)
Supplement: gkaf634_Supplemental_Files [file gkaf634_supplemental_files.zip › Table S1. Strain list.docx]

| *S. cerevisiae*: Strain MOY005  W303 *Mat a; ade2-1 ura3-1 leu2-3,112 his3-11,15 trp1-1 can1-100; [rho+]* | Gruschke et al., 2011 |
| --- | --- |
| *S. cerevisiae*: Strain MOY646 *nuc1Δ*  W303 *Mat α; ade2-1 ura3-1 leu2-3,112 his3-11,15 trp1-1 can1-100; nuc1::URA3; [rho+]* | Kehrein et al., 2015 |
| *S. cerevisiae*: Strain MOY082 *cbp3Δ*  W303 *Mat a; ade2-1 ura3-1 leu2-3,112 his3-11,15 trp1-1 can1-100; cbp3::KANMX4; [rho+]* | Gruschke et al., 2011 |
| *S. cerevisiae*: Strain MOY1847 *mrx4Δ*  W303 *Mat a; ade2-1 ura3-1 leu2-3,112 his3-11,15 trp1-1 can1-100; mrx4::LEU2; [rho+]* | This study |
| *S. cerevisiae*: Strain MOY1832 *cbp3Δ mrx4Δ*  W303 *Mat a; ade2-1 ura3-1 leu2-3,112 his3-11,15 trp1-1 can1-100; cbp3::kanMX4, mrx4::LEU2; [rho+]* | This study |
| *S. cerevisiae*: Strain MOY386 *cbp1Δ*  W303 *Mat a; ade2-1 ura3-1 leu2-3,112 his3-11,15 trp1-1 can1-100; cbp1::kanMX4; [rho+]* | [Gruschke et al., 2011](https://www.sciencedirect.com/science/article/pii/S1097276519308676?via%3Dihub#bib21) |
| *S. cerevisiae*: Strain MOY120 *cob::ARG8^m^*  W303 *Mat α; ade2-1 ura3-1 leu2-3,112 his3-11,15 trp1-1 CAN1; arg8::HIS3; cob::ARG8^m^; [rho+]* | [Gruschke et al., 2012](https://www.sciencedirect.com/science/article/pii/S1097276519308676?via%3Dihub#bib21) |
| *S. cerevisiae*: Strain MOY143 *cob::ARG8^m^ cbp3Δ*  W303 *Mat α; ade2-1 ura3-1 leu2-3,112 his3-11,15 trp1-1 CAN1; arg8::HIS3; cob::ARG8^m^; cbp3::kanMX4; [rho+]* | Gruschke et al., 2012 |
| *S. cerevisiae*: Strain MOY1828 *cob::ARG8^m^* *mrx4Δ*  W303 *Mat α; ade2-1 ura3-1 leu2-3,112 his3-11,15 trp1-1 CAN1; arg8::HIS3; cob::ARG8^m^; mrx4::LEU2; [rho+]* | This study |
| *S. cerevisiae*: Strain MOY1829 *cob::ARG8^m^* *cbp3Δ mrx4Δ*  *W303 Mat α; ade2-1 ura3-1 leu2-3,112 his3-11,15 trp1-1 CAN1; arg8::HIS3; cob::ARG8^m^; cbp3::kanMX4; mrx4::LEU2; [rho+]* | This study |
| *S. cerevisiae*: Strain MOY1720 *cob::ARG8^m^ cbs1Δ*  W303 *Mat α; ade2-1 ura3-1 leu2-3,112 his3-11,15 trp1-1 CAN1; arg8::HIS3; nuc1::URA3; cob::ARG8^m^; cbs1::kanMX4; [rho+]* | This study |
| *S. cerevisiae*: Strain MOY1830 *cob::ARG8^m^ cbs1Δ mrx4Δ*  W303 *Mat α; ade2-1 ura3-1 leu2-3,112 his3-11,15 trp1-1 CAN1; arg8::HIS3; nuc1::URA3; cob::ARG8^m^; cbs1::kanMX4; mrx4::LEU2; [rho+]* | This study |
| *S. cerevisiae*: Strain MOY1779 *cob::ARG8^m^* *cbs2Δ*  W303 *Mat α; ade2-1 ura3-1 leu2-3,112 his3-11,15 trp1-1 CAN1; arg8::HIS3; nuc1::URA3; cob::ARG8^m^*; *cbs2::kanMX4; [rho+]* | This study |
| *S. cerevisiae*: Strain MOY1960 *cob::ARG8^m^ cbs2Δ mrx4Δ*  W303 *Mat α; ade2-1 ura3-1 leu2-3,112 his3-11,15 trp1-1 CAN1; arg8::HIS3; nuc1::URA3; cob::ARG8^m^*; *cbs2::kanMX4; mrx4::LEU2; [rho+]* | This study |
| *S. cerevisiae*: Strain MOY532 *cob::ARG8^m^ bca1Δ*  W303 *Mat α; ade2-1 ura3-1 leu2-3,112 his3-11,15 trp1-1 CAN1; arg8::HIS3; cob::ARG8^m^*; *bca1::kanMX4; [rho+]* | [Gruschke et al., 2012](https://www.sciencedirect.com/science/article/pii/S1097276519308676?via%3Dihub#bib21) |
| *S. cerevisiae*: Strain MOY1831 *cob::ARG8^m^ bca1Δ mrx4Δ*  W303 *Mat α; ade2-1 ura3-1 leu2-3,112 his3-11,15 trp1-1 CAN1; arg8::HIS3; cob::ARG8^m^*; *bca1::kanMX4; mrx4::LEU2; [rho+]* | This study |
| *S. cerevisiae*: Strain MOY750 *cox1::ARG8^m^*  W303 *Mat α; ade2-1 ura3-1 leu2-3,112 his3-11,15 trp1-1 CAN1; arg8::HIS3; cox1::ARG8^m^; [rho+]* | This study |
| *S. cerevisiae*: Strain MOY1984 *cox1::ARG8^m^ mrx4Δ’*  W303 *Mat α; ade2-1 ura3-1 leu2-3,112 his3-11,15 trp1-1 CAN1; arg8::HIS3; cox1::ARG8^m^; mrx4::LEU2; [rho+]* | This study |
| *S. cerevisiae*: Strain MOY1985 *cox1::ARG8^m^* *mss51Δ*  W303 *Mat α; ade2-1 ura3-1 leu2-3,112 his3-11,15 trp1-1 CAN1; arg8::HIS3; cox1::ARG8^m^; mss51::LEU2; [rho+]* | This study |
| *S. cerevisiae*: Strain MOY1986 *cox1::ARG8^m^* *mss51Δ mrx4Δ*  W303 *Mat α; ade2-1 ura3-1 leu2-3,112 his3-11,15 trp1-1 CAN1; arg8::HIS3; cox1::ARG8^m^; mss51::LEU2; mrx4::hphMX6; [rho+]* | This study |
| *S. cerevisiae*: Strain MOY1987 *cox1::ARG8^m^ pet309Δ*  W303 *Mat α; ade2-1 ura3-1 leu2-3,112 his3-11,15 trp1-1 CAN1; arg8::HIS3; cox1::ARG8^m^; pet309::LEU2; [rho+]* | This study |
| *S. cerevisiae*: Strain MOY1988 *cox1::ARG8^m^ pet309Δ mrx4Δ*  W303 *Mat α; ade2-1 ura3-1 leu2-3,112 his3-11,15 trp1-1 CAN1; arg8::HIS3; cox1::ARG8^m^; pet309::LEU2; mrx4::hphMX6; [rho+]* | This study |
| *S. cerevisiae*: Strain MOY310 *cox2::COB cob::ARG8^m^* ¨  W303 *Mat a; ade2-1 ura3-1 leu2-3,112 his3-11,15 trp1-1 CAN1; arg8::HIS3; [rho+]* | Gruschke et al., 2012 |
| *S. cerevisiae*: Strain MOY1822 *cox2::COB cob::ARG8 ^m^ mrx4Δ*  W303 *Mat a; ade2-1 ura3-1 leu2-3,112 his3-11,15 trp1-1 CAN1; arg8::HIS3; cox2::COB cob::ARG8^m^; mrx4::LEU2; [rho+]* | This study |
| *S. cerevisiae*: Strain MOY479 *cox2::COB cob::ARG8 ^m^ qcr8Δ*  W303 *Mat a; ade2-1 ura3-1 leu2-3,112 his3-11,15 trp1-1 CAN1; arg8::HIS3; cox2::COB cob::ARG8^m^; qcr8::kanMX4; [rho+]* | Gruschke et al., 2012 |
| *S. cerevisiae*: Strain MOY1823 *cox2::COB cob::ARG8 ^m^ qcr8Δ mrx4Δ*  W303 *Mat a; ade2-1 ura3-1 leu2-3,112 his3-11,15 trp1-1 CAN1; arg8::HIS3; cox2::COB cob::ARG8^m^; qcr8::kanMX4; mrx4::URA3; [rho+]* | This study |
| *S. cerevisiae*: Strain MOY483 *cox2::COB cob::ARG8 ^m^ cyt1Δ*  W303 *Mat a; ade2-1 ura3-1 leu2-3,112 his3-11,15 trp1-1 CAN1; arg8::HIS3; cox2::COB cob::ARG8^m^; cyt1::kanMX4; [rho+]* | Gruschke et al., 2012 |
| *S. cerevisiae*: Strain MOY1824 *cox2::COB cob::ARG8 ^m^ cyt1Δ mrx4Δ*  W303 *Mat a; ade2-1 ura3-1 leu2-3,112 his3-11,15 trp1-1 CAN1; arg8::HIS3; cox2::COB cob::ARG8^m^; cyt1::kanMX4; mrx4::URA3; [rho+]* | This study |
| *S. cerevisiae*: Strain MOY368 *cox2::COB cob::ARG8 ^m^ rip1Δ*  W303 *Mat a; ade2-1 ura3-1 leu2-3,112 his3-11,15 trp1-1 CAN1; arg8::HIS3; cox2::COB cob::ARG8^m^; rip1::kanMX4; [rho+]* | Gruschke et al., 2012 |
| *S. cerevisiae*: Strain MOY1825 *cox2::COB cob::ARG8 ^m^ rip1Δ mrx4Δ*  W303 *Mat a; ade2-1 ura3-1 leu2-3,112 his3-11,15 trp1-1 CAN1; arg8::HIS3; cox2::COB cob::ARG8^m^; rip1::kanMX4; mrx4::URA3; [rho+]* | This study |
| *S. cerevisiae*: Strain MOY1609  W303 *Mat a; ade2-1 ura3-1 leu2-3,112 his3-11,15 trp1-1 can1-100; cbp3::kanMX4; pRS403-EcYRS^pBpa^-tRNA^CUA^::HIS3; [rho+]* | Ndi et al., 2019 |
| *S. cerevisiae*: Strain MOY1956  W303 *Mat a; ade2-1 ura3-1 leu2-3,112 his3-11,15 trp1-1 CAN1; cbp3::kanMX4; pRS403-EcYRS^pBpa^-tRNA^CUA^::HIS3; mrx4::LEU2; [rho+]* | This study |
| *S. cerevisiae*: Strain MOY1224 Cbp3-BirA*  W303 *Mat α; ade2-1 ura3-1 leu2-3,112 his3-11,15 trp1-1 can1-100; nuc1::URA3; CBP3-BirA*::HIS3; [rho+]* | Salvatori et al., 2020 |
| *S. cerevisiae*: Strain MOY1856 Cbp3-BirA* *mrx4Δ*  W303 *Mat α; ade2-1 ura3-1 leu2-3,112 his3-11,15 trp1-1 can1-100; nuc1::URA3; CBP3-BirA*::HIS3; mrx4::LEU2; [rho+]* | This study |
| *S. cerevisiae*: Strain MOY1293 Cbs1-BirA*  W303 *Mat α; ade2-1 ura3-1 leu2-3,112 his3-11,15 trp1-1 can1-100; nuc1::URA3; CBS1-BirA*::HIS3; [rho+]* | Salvatori et al., 2020 |
| *S. cerevisiae*: Strain MOY1857 Cbs1-BirA* *mrx4Δ*  W303 *Mat α; ade2-1 ura3-1 leu2-3,112 his3-11,15 trp1-1 can1-100; nuc1::URA3; CBS1-BirA*::HIS3; mrx4::LEU2; [rho+]* | This study |
| *S. cerevisiae*: Strain MOY1294 Cbs2-BirA*  W303 *Mat α; ade2-1 ura3-1 leu2-3,112 his3-11,15 trp1-1 can1-100; nuc1::URA3; CBS2-BirA*::HIS3; [rho+]* | Salvatori et al., 2020 |
| *S. cerevisiae*: Strain MOY1858 Cbs2-BirA* *mrx4Δ*  W303 *Mat α; ade2-1 ura3-1 leu2-3,112 his3-11,15 trp1-1 can1-100; nuc1::URA3; CBS2-BirA*::HIS3; mrx4::LEU2; [rho+]* | This study |
| *S. cerevisiae*: Strain MOY1242 Kgd4-BirA*  W303 *Mat α; ade2-1 ura3-1 leu2-3,112 his3-11,15 trp1-1 can1-100; nuc1::URA3; KGD4-BirA*::HIS3; [rho+]* | Salvatori et al., 2020 |
| *S. cerevisiae*: Strain MOY1955 Kgd4-BirA* *mrx4Δ*  W303 *Mat α; ade2-1 ura3-1 leu2-3,112 his3-11,15 trp1-1 can1-100; nuc1::URA3; KGD4-BirA*::HIS3; mrx4::LEU2; [rho+]* | This study |
| *S. cerevisiae*: Strain MOY1968 Cbp3-ALFA  W303 *Mat a; ade2-1 ura3-1 leu2-3,112 his3-11,15 trp1-1 can1-100; nuc1::URA3; CBP3-ALFA::TRP1; [rho+]* | This study |
| *S. cerevisiae*: Strain MOY1976-1978 Mrx4-GFP-ALFA  W303 *Mat a; ade2-1 ura3-1 leu2-3,112 his3-11,15 trp1-1 can1-100; MRX4-GFP-ALFA::TRP1; [rho+]* | This study |
| *S. cerevisiae*: Strain MOY1979-1981 *cob::ARG8^m^* Mrx4-GFP-ALFA  W303 *Mat α ade2-1 ura3-1 leu2-3,112 his3-11,15 trp1-1 CAN1; arg8::HIS3; cob::ARG8^m^ MRX4-GFP-ALFA::TRP1; [rho+]* | This study |
| *S. cerevisiae:* Strain MOY2246 Mrps17-3xFLAG  W303 *Mat α; ade2-1 ura3-1 leu2-3,112 his3-11,15 trp1-1 can1-100; nuc1::URA3; MRPS17-3xFLAG::TRP1; [rho+]* | This study |
| *S. cerevisiae*: Strain MOY2377 Cbp3-3xFLAG  W303 *Mat α; ade2-1 ura3-1 leu2-3,112 his3-11,15 trp1-1 can1-100; nuc1::URA3; CBP3-3xFLAG::TRP1; [rho+]* | This study |
| *S. cerevisiae*: Strain YSC052 Mrps17-3xFLAG  S288C *Mat a; GAL2 HAP1 MIP1[S] G1981A ura3Δ0  mrps17::MRPS17-3xFLAG; [rho+]* | Couvillion et al., 2016 |
| *S. cerevisiae*: Strain YSC061 Cbs1-3xFLAG  S288C *Mat a; GAL2 HAP1 MIP1[S] G1981A ura3Δ0  mrps17::MRPS17-3xHA cbs1::CBS1-3xFLAG; [rho+]* | This study |
| *S. cerevisiae*: Strain YSC060 Cbs2-3xFLAG  S288C *Mat a; GAL2 HAP1 MIP1[S] G1981A ura3Δ0  mrps17::MRPS17-3xHA cbs2::CBS2-3xFLAG; [rho+]* | This study |
| *S. cerevisiae*: Strain YSC374 Cbs1-3xFLAG *mrx4Δ*  S288C *Mat a; GAL2 HAP1 MIP1[S] G1981A ura3Δ0  mrps17::MRPS17-3xHA cbs1::CBS2-3xFLAG mrx4::kanMX; [rho+]* | This study |
| *S. cerevisiae*: Strain YSC378 Mrps17-3xFLAG *mrx4Δ*  S288C *Mat a; GAL2 HAP1 MIP1[S] G1981A ura3Δ0  mrps17::MRPS17-3xFLAG mrx4::kanMX6; [rho+]* | This study |
| *S. cerevisiae*: Strain YSC380 Mrx4-3xFLAG  S288C *Mat a; GAL2 HAP1 MIP1[S] G1981A ura3Δ0  mrps17::MRPS17-3xHA mrx4::MRX4-3xFLAG; [rho+]* | This study |
